# Association between cardiorespiratory fitness and metabolic risk factors in a population with mild to severe obesity

**DOI:** 10.1186/s40608-018-0183-7

**Published:** 2018-01-31

**Authors:** Kathy Do, Ruth E. Brown, Sean Wharton, Chris I. Ardern, Jennifer L. Kuk

**Affiliations:** 1 School of Kinesiology and Health Science, York University, School of Kinesiology and Health Science, Toronto, M3J 1P3 Canada; 2 The Wharton Medical Clinic, Toronto, ON Canada

**Keywords:** Cardiorespiratory fitness, Metabolic risk factors, Obesity

## Abstract

**Background:**

Previous literature suggests the beneficial effects of fitness on abdominal obesity may be attenuated in obesity and abolished in severe obesity. It is unclear whether the beneficial association between fitness and health is similarly present in those with mild and severe obesity.

**Methods:**

Patients from the Wharton Medical Clinic (*n* = 853) completed a clinical examination and maximal treadmill test. Patients were categorized into fit and unfit based on age- and sex-categories and body mass index (BMI) class (mild: ≤ 34.9 kg/m^2^, moderate: 35–39.9 kg/m^2^ or severe obesity: ≥ 40 kg/m^2^).

**Results:**

Within the sample, 41% of participants with mild obesity had high fitness whereas only 25% and 11% of the participants with moderate and severe obesity, respectively, had high fitness. BMI category was independently associated with most of the metabolic risk factors, while fitness was only independently associated with systolic blood pressure and triglycerides (*P* < 0.05). The prevalent relative risk for pre-clinical hypertension, hypertriglyceridemia and hypoalphalipoproteinemia and pre-diabetes was only elevated in the unfit moderate and severe obesity groups (*P* < 0.05), and fitness groups were only significantly different in their relative risk for prevalent pre-clinical hypertension within the severe obesity group (*p* = 0.03). High fitness was associated with smaller waist circumferences, with differences between high and low fitness being larger in those with severe obesity than mild obesity (Men: *P* = 0.06, Women: *P* = 0.0005).

**Conclusions:**

Thus, in contrast to previous observations, the favourable associations of having high fitness and health may be similar if not augmented in individuals with severe compared to mild obesity.

## Background

The fit-fat paradox has been the topic of investigation for many years [1–5]. Specifically, it is suggested that individuals with mild obesity and a high fitness may not present with the typically expected negative health factors associated with obesity and may have lower risk of mortality than their normal-weight unfit counterparts [1–5]. The health benefits of having a high fitness are thought to be in part due to reduced visceral obesity for a given body mass index (BMI) [6]. However, the decreased abdominal obesity associated with a high fitness may be attenuated with higher levels of obesity, and it is suggested that there may no differences in abdominal obesity by fitness in individuals with moderate and severe obesity [7]. Conversely, the potential beneficial associations between high fitness and health may be larger in severe obesity than milder obesity groups [6, 8], perhaps due to the greater deterioration in health commonly observed in severe obesity classes. Populations with moderate to severe obesity have increased in prevalence and have reached over 5% in Canada [9] but remain a relatively understudied population. Thus, the primary objective of this study is to determine the relationships between fitness and metabolic risk factors in individuals with higher levels of obesity.

## Methods

### Participants

The sample included 853 patients with obesity who attended the Wharton Medical Clinic. Participants were included if they underwent measurements of blood pressure, general blood work and a standardized treadmill test during their first 3 months of enrolling at the weight management centre. All participants provided written informed consent knowing their decision to participate would not alter the care provided and that they could withdraw consent at any time. York University Institutional Review Board approved the study protocol used (Certificates: 2013–123 and #e2017–166). The datasets generated and analyzed during the current study are not publicly available due to privacy laws associated with medical data but are available with a data sharing agreement as approved by the relevant institutional ethics committee and the health information custodian (S. Wharton).

### Clinical examination

Blood samples were obtained via venipuncture after at least an 8 h fast to assess triglycerides, glucose, and high density lipoprotein (HDL) cholesterol using standard procedures by certified medical laboratories. Blood pressure was measured manually at the clinic by trained technicians. Preclinical hypertension was defined as blood pressure ≥ 130/85 mmHg or use of hypertensive medications. Preclinical hypertriglyceridemia was defined as triglyceride > 1.7 mM or use of lipid medications. Preclinical hypoalphalipoproteinemia was defined as HDL levels less than 1.0 mM in men or 1.3 mM in women or use of lipid medications. Prediabetes was defined as glucose ≥ 5.6 mM or use of diabetes medications. Waist circumference was measured at the midpoint between the superior iliac spine and lowest rib [10]. BMI was used to categorize obesity levels: mild: BMI 30–24.9 kg/m^2^, moderate: BMI 35–39.9 kg/m^2^ and severe: BMI ≥ 40 kg/m^2^ [11].

Maximal oxygen uptake (VO_2max_) was estimated from a graded multistage treadmill Bruce protocol. Treadmill time was used to predict VO_2max_ using sex-specific equations [12, 13]. Participants were stratified by fitness based on standard age- and sex- specific VO_2max_ cutoffs: unfit (< 20th percentile) or fit (≥ 20th percentile) [14].

### Statistical analysis

ANOVA and chi-squared tests were used to determine group differences in participant characteristics. Differences in health risk between mild, moderate or severe obesity groups by fitness groups were assessed using generalized linear models with obesity class (continuous term) and fitness main effect and interaction terms adjusting for age, sex and relevant medication use (diabetes, lipid or blood pressure). When the interaction term was not significant, the model was re-run without the interaction term. When the interaction or obesity group and/or fitness main effect was observed, the least squared means were reported for the obesity-fitness groups with least squared difference post hoc tests to assess group differences.

The relative risk for prevalent preclinical risk factors for the obesity and fitness groups was assessed using main effects and interaction terms adjusting for age and sex using the method proposed by Zou [15]. When the interaction term was not significant, the model was re-run without the interaction term. When the interaction or obesity and/or fitness main effect was observed, a least squared difference post hoc was conducted to assess group differences. Analyses were performed using SAS v9.4 (SAS Institute, Cary, NC).

## Results

Within the sample, 41% of participants with mild obesity were fit whereas only 25% and 11% of the participants with moderate and severe obesity, respectively, were considered fit (Table 1). Individuals with high fitness tended to be younger (47.6 vs 51.4 years), had a lower BMI (35.8 vs. 41.1 kg/m^2^) and more likely to be female (84.4 vs 75.9%, *P* < 0.05).

**Table 1:** Participant characteristics stratified by BMI group and fitness

	Mild Obesity		Moderate Obesity		Severe Obesity	
	Fit	Unfit	Fit	Unfit	Fit	Unfit
N	107	151	60	181	38	316
Age (y)	51.0(10.5)^bce^	54.8(11.0)^acef^	43.9(11.2)^abdf^	53.5(11.9)^cef^	44.0(11.9)^abdf^	48.5(11.4)^b-e^
Sex (%female)	80.4	75.5	90.0	77.9	86.8	75.0
BMI (kg/m^2^)	32.2(1.6)^b-f^	32.5(2.0)^c-f^	37.2(1.5)^abef^	37.4(1.4)^abef^	43.7(3.2)^a-d,f^	47.4(6.6)^a-e^
VO_2max_ (ml/kg/min)	36.6(4.1)^bdf^	21.6(6.1)^ace^	35.6(1.7)^bdf^	20.5(6.1)^ace^	35.6(3.1)^bdf^	20.4(6.2)^a-c,e^
Glucose (mM)	5.7(1.4)^f^	5.8(1.3)^f^	5.7(1.4)^f^	6.0(1.3)^f^	5.8(1.1)^f^	6.3(1.9)^a-e^
HDL (mM)	1.3(0.4)^cdf^	1.4(0.4)^cdf^	1.2(0.3)^ab^	1.2(0.3)^ab^	1.3(0.3)	1.2(0.3)^ab^
Triglycerides (mM)	1.4(0.9)^df^	1.5(0.9)^f^	1.4(0.9)^f^	1.7(0.8)^a^	1.5(0.9)	1.8(1.0)^a-c^
SBP (mmHg)	125(12)^df^	128(14)^af^	125(13)^df^	131(13)^a,c,f^	128(12)^f^	135(14)^a-e^
DBP (mmHg)	78(7)^df^	79(8)^f^	78(7)^df^	80(10)^acf^	81(10)^f^	83(9)^a-e^
T2D Med (%)	12.1^df^	18.5	18.3	23.2^a^	18.4	25.9^ae^
BP Med (%)	31.8^d,f^	40.4^d,f^	28.3^d,f^	55.8^a-c,e^	23.7^d,f^	55.1^a-c,e^
Lipid Med (%)	30.8	37.1^c^	21.7^bdf^	40.9^c,e^	21.1^df^	37.7^c,e^

Mild Obesity (BMI: 30–34.9 kg/m^2^); Moderate obesity (BMI: 35–39.9 kg/m^2^); Severe obesity (BMI: ≥ 40 kg/m^2^). Fit: top 80th percentile for age and sex categories

*BMI* body mass index, *VO*_*2max*_ maximal oxygen consumption, *HDL* high density lipoprotein, *SBP* systolic blood pressure, *DBP* diastolic blood pressure, *T2D* type 2 diabetes

^a^Significantly different compared to fit-mild obesity (*P* < 0.05)

^b^Significantly different compared to unfit-mild obesity (*P* < 0.05)

^c^Significantly different compared to fit-moderate obesity (*P* < 0.05)

^d^Significantly different compared to unfit-moderate obesity (*P* < 0.05)

^e^Significantly different compared to fit-severe obesity (*P* < 0.05)

^f^Significantly different compared to unfit-severe obesity (*P* < 0.05)

**Fig. 1:**
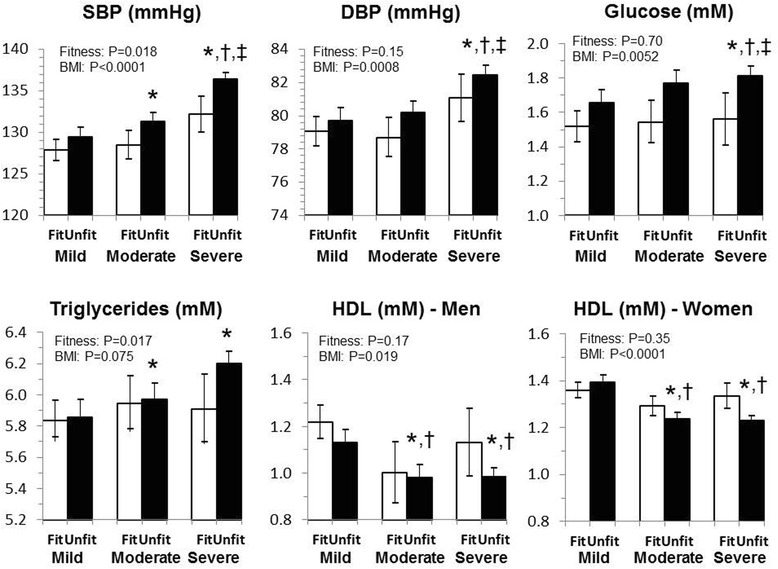
Least squared adjusted means for blood pressure, glucose, triglycerides and HDL cholesterol in individuals by obesity-fitness group. *Significantly different from the fit category within BMI category; †Significantly different from mild obesity within fitness category; ‡Significantly different from moderate obesity within fitness category. No significant differences by fitness within BMI category BMI, body mass index; SBP, systolic blood pressure; DBP, diastolic blood pressure; HDL, high density lipoprotein. Models are adjusted for age, sex and relevant blood pressure, diabetes or lipid medication use

The mean blood pressure, glucose, triglycerides and HDL adjusted by age and sex stratified by BMI and fitness category are shown in Fig. 1. As expected, BMI category was independently associated with most of the metabolic risk factors, while fitness was independently associated with SBP and triglycerides (*P* < 0.05). There were no significant differences between fitness groups within a BMI category (*P* > 0.05). Further, there were no significant differences between obesity groups within the fit category for any of the metabolic variables (*P* < 0.05). Unfit, severe obesity groups had significantly worse metabolic profiles as compared to the fit mild obesity group (*P* < 0.05) and the unfit mild obesity group (except triglycerides, *P* < 0.05).

**Fig. 2:**
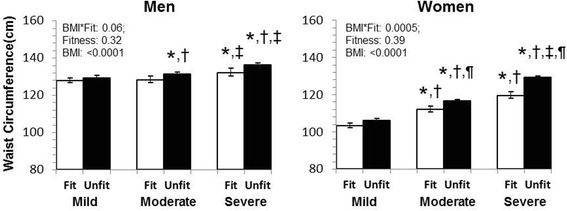
Least squared adjusted means for waist circumference in men and women by obesity-fitness group. *Significantly different from the fit category within BMI category; †Significantly different from mild obesity within fitness category; ‡Significantly different from moderate obesity within fitness category. ¶Significantly different from the fit category within BMI category Models are adjusted for age

Those with low fitness and higher obesity tended to have a larger waist circumference (Fig. 2). There was a significant BMI x fitness interaction, indicating that the differences in waist circumference between fitness groups were greater in those with higher obesity classes in men (*P* = 0.06) and women (*P* = 0.0005). However, the difference in waist circumference between fitness groups only attained significance in the moderate (Fit versus Unfit: 112.1 versus 116.5 cm, *P* = 0.001) and severe (Fit versus Unfit: 119.6 versus 129.2 cm, *P* < 0.0001) obesity groups in women.

**Fig. 3:**
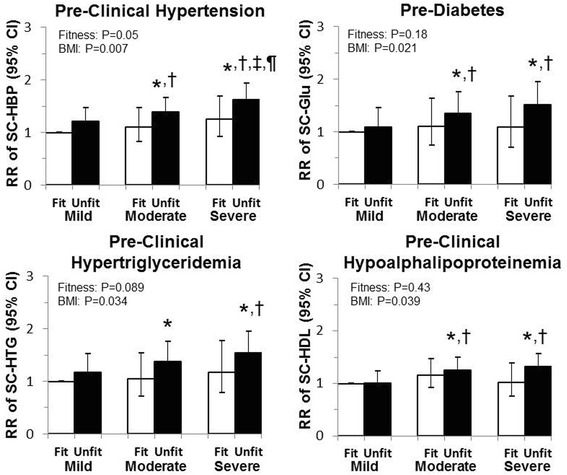
Relative risk for preclinical hypertension, diabetes and hypertriglyceridemia and hypoalphalipoproteinemia in men and women by obesity-fitness group. *Significantly different from the fit category within BMI category; †Significantly different from mild obesity within fitness category; ‡Significantly different from moderate obesity within fitness category. ¶Significantly different from the fit category within BMI category. Models are adjusted for age and sex

The prevalent relative risk for pre-clinical hypertension, hypertriglyceridemia and hypoalphalipoproteinemia and pre-diabetes was only elevated in the unfit moderate and severe obesity groups as compared to fit and unfit groups with mild obesity (Fig. 3, *P* < 0.05). The only difference between fitness groups within a given BMI category was in the relative risk for prevalent pre-clinical hypertension within the severe obesity group (Fit versus Unfit RR: 0.77, 0.59–0.99, *p* = 0.047).

## Discussion

To our knowledge, this is the first study to demonstrate that the association between fitness and health may be similar if not augmented in individuals with severe obesity as compared to mild obesity and extends previous research done in populations with normal weight, overweight and mild obesity. Further, we suggest that these differences in health risk by fitness may be related with differences in waist circumference.

High fitness is commonly defined in the health literature as being in the top 80% of an age- and sex-category as this is the threshold often reported as being associated with the greatest gains in health benefits [1, 2]. This means that the 10% prevalence of high fitness observed in those with severe obesity is 8 times lower than what would be expected in the general population. Within the present study, fitness was predicted using symptom-limited treadmill testing. Therefore, some individuals may have stopped due to problems such as musculoskeletal pain which is shown to be more likely in populations with obesity [16] rather than cardiovascular fatigue. Also, certain medications that are more prevalent in populations with obesity, such as beta blockers, can influence heart rate and blood pressure and increase the likelihood of experiencing early cardiovascular fatigue during exercise [17]. Given that our sample comprised individuals with higher levels of obesity who are likely to have more health problems, our ability to accurately identify patients with high fitness may have been reduced. Nevertheless, the concept that an individual can present with high fitness despite severe obesity is a novel observation that has important clinical and public health implications.

Several studies have demonstrated a positive association between obesity and metabolic risk factors, as well as an inverse association between fitness and metabolic risk [2, 3]. These observations are largely limited to populations consisting of mainly normal weight, overweight and mild obesity [1, 2]. Borodulin et al. [8] and Lee et al. [6] demonstrated that there was a stronger association between CRF and systolic blood pressure with increasing levels of adiposity. Conversely, data from the Aerobics Centre Longitudinal Study suggest that the association between fitness and blood pressure may be weaker in those with greater obesity [18]. We extend these findings to demonstrate that the benefits of fitness for most of the metabolic risk factors are similar for all obesity classes. The lone exception was that those with severe obesity, individuals who were fit were less likely to have pre-clinical or clinical hypertension than those were unfit.

The health effects of fitness are suggested to be mediated in part through the positive health benefits of engaging in regular physical activity [2]. Physical activity has been shown to improve fasting glucose by increasing the rate of glucose uptake in skeletal muscle [19] and improve lipid metabolism through increases in lipoprotein lipase in both skeletal muscle and adipose tissue [20]. As individuals with severe obesity are more likely to have deteriorations in these metabolic risk factors, it may not be surprising that the benefits of fitness may also extend to those with severe obesity. Nevertheless, in our study and others [18, 21, 22], obesity was more strongly associated with metabolic health risk than fitness. However, it should be of note that within the fit individuals, those with severe obesity did not have significantly elevated glucose, blood pressure or lipids as compared to those with mild obesity. This supports the potentially important health benefits of having a high fitness level, particularly for those with severe obesity.

The beneficial effects of fitness on health may also be attributed to differences in abdominal obesity. Wong et al. [5] report that in men the differences in visceral obesity with high fitness are reduced with increasing BMI and may be completely abolished at a BMI of ≥35 kg/m^2^. This would suggest that the benefits of fitness should be attenuated with those with moderate and severe obesity. However, our results in women and to a lesser degree in men, demonstrate that there was a greater difference in waist circumference between fitness groups in the moderate and severe obesity group than the mild obesity group: an observation that mirrors our results for metabolic health. These differences may in part steam from the sex or other demographic differences between the ACLS and WMC populations studied, but may suggest that fitness may be particularly important for women with obesity. However, these primary observations need further investigation for confirmation.

The strengths and limitations of our study warrant mention. Although this study used a larger sample size with higher levels of obesity than previous studies, the cross sectional design of our study does not allow us to infer causality. Also, we were unable to adjust for other factors such as physical activity, ethnicity, smoking status, and education as these variables were not consistently reported within our clinical population.

## Conclusions

With the increasing prevalence of obesity [23], efforts to understand variation in health risk within this population are of considerable public health importance. We demonstrate that the benefits of fitness on metabolic health appear similar if not augmented in those with higher levels of obesity as compared to those with lower levels of obesity. Thus, it may be equally if not more important to promote physical activity and fitness behaviours to this increasingly prevalent group of individuals in order to obtain metabolic health benefits.
